# Social relationship changes in victim families due to a social disaster: Experiences of student victims’ families in the South Korean Sewol ferry disaster

**DOI:** 10.1371/journal.pone.0188699

**Published:** 2017-12-07

**Authors:** Sun Mi Cho, Ansuk Jeong, Jung Hee Ha, Eun Young Kim

**Affiliations:** 1 Department of Psychiatry, School of Medicine, Ajou University, Kyunggi-Do, South Korea; 2 Department of Psychology, The University of Utah Asia Campus, Incheon, South Korea; 3 Graduate School of Counseling Psychology, Hanyang University, Seoul, South Korea; 4 Department of Education, Kyungpook National University, Daegu, South Korea; The Chinese University of Hong Kong, HONG KONG

## Abstract

The Sewol ferry incident on April 16, 2014 in South Korea claimed the lives of 304 individuals, including about 250 high school students on a school trip. The majority of South Korean citizens were watching live updates on the capsized Sewol ferry, anxiously watching on TV how the vessel fully sunk over time. They were desperately hoping for the rescue of the survivors inside. However, their anxiety had become shock, anger, and helplessness, and the disaster has become a daunting, collective trauma, not just to the victims and their families, but also to the citizens who were exposed only through the media. In this study, we interviewed victims’ families two years after the incident. We explored how they have experienced changes in their social relationships. We conducted semi-structured interviews of 54 family members of the student victims. We qualitatively examined the data applying a thematic analysis. Changes in their social relationships were largely divided into the relationships in the proximal environment and the relationships in distal environments. The former included subcategories such as immediate family, coworkers, friends, relatives, survived students and their parents, and concepts corresponding to each subcategory. The latter involved subcategories such as neighbors, other citizens, the victims’ family committee, government, and society, and concepts subject to each subcategory. Based on these findings, rehabilitation plans for trauma victims and their families should take into account the significant changes in their social relationships and the further consequences of those changes.

## Introduction

The sinking of the Sewol ferry, also referred to as the 416 Sewol ferry disaster, occurred in the South Korean waters near Jeollanamdo on April 16, 2014. The ferry was carrying 476 people, including passengers and crew members. Most of the passengers were students from Danwon high school who were on a school trip. Of the 325 students on board, 250 died or went missing, and only 75 survived. The captain and most of the crew fled the ship after it capsized; thus, no proper evacuation orders were issued. Investigations revealed that unsafe operation by Chonghaejin Marine Company Ltd., the lack of disaster countermeasures, and the government’s delayed response were also main causes of the disaster. Yet, it should be noted that delayed rescue operation by the Korean Coast Guard and the Ministry of Oceans and Fisheries, in particular, and subsequent bureaucratic and political cover-ups exacerbated the situation [1]. The Sewol ferry disaster was a social disaster that can be regarded as caused by incompetence and fatal errors of social or socio-technical systems [2]. The accident claimed the lives of 304 individuals, including more than 200 high school students on a school trip. The wreck of the Sewol seemed also to traumatize citizens who were merely exposed through media, maybe because the tragedy appeared to have been so preventable.

Generally, a disaster is a condition where damage or loss of life is too massive for humans’ capacity to cope [3], and such an event can devastate the affected individuals and further extend its damage to corresponding and nearby areas [4]. A disaster can vary in its magnitude, extent, and further impact. It is generally divided into two categories: a natural disaster and a manmade disaster. While the former covers natural hazards such as earthquakes, floods, droughts, or landslides, the latter corresponds to: i) engineering or technological hazards such as the collapse of bridges and buildings, industrial accidents, or transport crashes; and, ii) purposeful incidents such as massacres, terrorist acts, or wars [5]. The social impact of a disaster is generally affected by if it is a natural or manmade disaster. A natural disaster and a manmade disaster may occur simultaneously and interact with each other in various ways [6]. Manmade disasters seem to result in far more severe consequences than natural disasters [7]. If a disaster’s causes can be attributed to certain individuals or a particular group of people, the fear and anger of the affected people will be directed toward them. Victims and their families may suffer from strong emotional distress and may have difficulty dealing with rage, distrust, self-criticism, or guilt, eventually resulting in severe mental health problems in the long-term [7–9].

A social disaster can impact the victims’ social relationships as well as their mental health. Picou and Marshall found, in their study of damage by a hurricane in 2005, that the victims not only went through loss of family and homes but also were exposed to suffering from discrimination, conflict, and contempt during restoration [10]. When they were relocated to a new site, the victims had to endure distrust and conflict with their hosts and adjacent communities. Similarly, it was reported that after a disaster, victims were exposed to rumors, competition, and conflicts that exacerbated their anxiety and fear [11], generational conflicts [12], and significantly weakened communal bonds [13]. For the individuals affected by a social disaster, damage can be extensive and prolonged and can result in various social adjustment problems, at times undermining existing social systems and cultural structure. Therefore, it is vital to account for significant changes in social relationships when a disaster happens, as well as for the consequences of those changes (e.g., secondary trauma) [14].

However, existing research on social disasters has mainly studied psychological trauma and symptoms, overlooking issues of social relationship change for victims. Moreover, existing studies have rarely addressed victims’ interpersonal experiences or changes in social relatedness in their daily life by looking at their own voices. Looking into victims’ internal experiences helps develop effective interventions for them [15], and a qualitative approach can suitably inform understanding their own perspectives regarding such specific situations [16]. Also, given that the loss of a child can result in mourning far more intense and prolonged than any other loss [17–18], we can assume that victims’ families’ experiences in the Sewol disaster may show different characteristics compared to those from other social disasters. In this study, we adopted a qualitative approach to grasp changes in social relationships. For affected individuals, trauma is not a mere set of events; it can be perceived differently depending on how they make sense of the incident individually and socially, and how they react to it [5].

This study is intended to examine how the families’ social experiences have changed since the tragedy. We conducted detailed interviews with family members of the Danwon high school student victims, exploring how their social relationships have changed since the Sewol ferry disaster. Looking into their subjective experiences can contribute to the establishment of effective and realistic interventions for victims and their families through the development of proper coping and social support resources as well as of relevant rehabilitation policies.

## Methods

### Participants

Study participants were immediate family members, mostly parents, of the high school student victims. If a student victim had lived with a grandparent or other relative as a caretaker, he or she was also included in the study. The interviews were conducted from February to June 2016. The committee for special investigation of the Sewol ferry disaster provided a register of the student victims’ families who had signed a consent form for release of information for research purposes. The committee and this study’s research team contacted the 127 families who consented, and 64 participants agreed to participate in the interviews. While preparing for the interviews, 10 participants left the study due to scheduling conflicts or personal issues. We conducted semi-structured interviews with the remaining 54 participants, comprising of 27 fathers, 24 mothers, 2 grandparents, and 1 aunt. The interviews lasted from 2 to 2 ½ hours.

### Participants interview guide development

We developed a first set of in-depth interview questions based on: i) reports of physical and mental health of the Sewol victims and their families, reports on human rights issues regarding the 416 Sewol disaster, and other pertinent materials, and ii) results from a pilot interview with four student victims’ families. The in-depth interview questions largely consisted of an introduction, interview content, and an ending. The introduction covered the purpose of the interview, introducing the interviewer, signing a written consent form, explaining about recording and follow-ups, and offering a gift as compensation. The interview content included questions about aid and support provided by the government, organizations, and individuals, their social relationships from April 15, 2014 to the current date, and lastly changes in family relationships and other relationships outside the family. This set of interview questions was revised two times through discussions among the researchers. A pilot study was conducted with the revised set of questions. After interviewers and researchers, via two meetings, revised and modified the in-depth interview, based on the pilot study results, the final set of in-depth interview questions was produced.

### Interview procedure

The research team consisted of four researchers with doctoral degrees in counseling or clinical psychology, one researcher with a master’s degree, and one with a bachelor’s degree. In addition to the research team, 10 more interviewers joined to conduct the in-depth interviews. All interviewers were female and had a registered license in counseling or clinical psychology with at least three years of clinical experience working at a public health organization or community mental health center. We performed each interview based on the set of in-depth questions to establish consistency and reliability across interviewers and to prevent response bias from face-to-face interactions between interviewees and interviewers. We implemented an education and training plan for interviewers. We provided educational meetings and several training sessions to interviewers two months prior to the actual interviews. An interview manual summarizing a re-questioning technique in the event of uncertain responses and a technique for conducting follow-up questions was introduced and used to maintain consistency and accuracy across interviewers in obtaining interview content. This study, as qualitative research, has fully followed the guidelines suggested by Lincoln and Guba [16], consisting of truth value, applicability, consistency, and neutrality. Before launching the project, this study was approved by the Ajou University Medical Center Institutional Review Board (SBR-SUR-15-519, SBR-SUR-15-520). We prepared a secure place in Ansan [The high school is located in Ansan][15] convenient for participants’ access to the interviews. The interviewers scheduled the interviews with each participant by phone. During these phone conversations, the participants were told the reasons and purpose for the interview and the qualifications of the interviewers, and were given an opportunity to ask questions. During the introduction, a written consent form was obtained regarding audio recordings and follow-up interviews when necessary. Accordingly, if specific information was found to be missing after the interviews, the interviewers arranged meetings for additional interviews or contacted the participants by phone to obtain the missing information. The semi-structured interviews based on the pre-established set of questions were performed, allowing additional questions whenever needed. Two participants were severely distressed during the interview; after obtaining their consent, they were referred to a nearby mental health center. Compensation was considered for their time and effort for the interview and a gift was offered as approved by IRB.

### Analysis

This study aimed to draw common themes and implications from interview content, rather than to apply concepts or hypotheses from existing theories or studies for analysis. To grasp changes in the relationships established prior to the disaster, the victims’ families were asked about such changes in their immediate family and other relationships. The research team attempted to draw themes and concepts based on the families’ responses to those questions. This method is useful to capture meanings and subjective experiences involving participants’ perceptions, understandings, and conceptions about themselves and their surroundings under certain circumstances. For this, a complete transcription of each in-depth interview was performed and cross-checked so that the participants’ expressions and thoughts were accurately transcribed. Then, the interview data were sorted by research questions. Applying a thematic analysis method [19], we attempted to identify recurring, meaningful themes and grouped categories and concepts emerging out of the raw data. We applied a coding process without attempting to fit the data into any pre-established frame, so that the identified items were solely data-driven. A thematic analysis method searches for themes, focusing on similarities and differences among the participant’s experiences and their meanings. This exploration seeks to ‘discover’ new themes for certain situations. Through this process, we aimed to capture in-depth meanings, perceptions, and the various feelings that the student victims’ families had been experiencing.

Five researchers with doctoral degrees in psychology with previous research experience in thematic analysis and two researchers with master’s degrees participated in a thematic analysis of the responses to the interview questions. Seven researchers separately reviewed and sorted the data according to each research question, and performed coding independently. As each analysis was completed, all researchers gathered to discuss coding differences; for three months, the researchers worked out coding disagreements. A unified codebook was finalized after five modifications. Based on the codebook, the researchers recoded the interview data. For each item in the codebook, corresponding interview content from the data was written down. Similar content was regrouped and re-sorted repetitively via comparison analysis. From these processes, the raw data was summarized and trimmed to 15 pages, which would serve as the basic materials for interpretations. A frame of categories, subcategories, and concepts was established, and the details of the contents were composed. The researchers examined whether the frame and the content were valid and finalized the frame for interpretations.

## Results

### Changes in social relationships

#### Relationships with their immediate family members

The student victims’ families stated that they have experienced significant changes in their relationships with their immediate family members in daily life. The most salient change was seen in communication, with either sudden decreases or increases in conversation. For example, some had barely talked to one other, avoiding mentioning anything related to the Sewol incident. And they no longer celebrated holidays or anniversaries together:

We rarely have time together for supper. You mean, together with family. When ** was with us, we always had supper together. Since ** ‘s gone, we have never gone out for dinner or something. His sister is now in college and comes home on Fridays. Yet, we don’t talk like before, laughing. She’s just in her room looking at her cell phone. We don’t talk any more. Years ago, we used to go out together at least once or twice a month or drive somewhere near just to talk and eat. But, we don’t do that anymore. We rarely go out together for dinner or something (A 49-year-old father who lost a daughter).

Additionally, the families reported that they had become sensitive to other family members’ psychological changes and had experienced changes in their families’ roles or structures. They often felt that some family members, such as children or old grandparents, were being neglected. Parents realized that the siblings of the lost child were experiencing certain changes in their own lives. For example, some parents were aware that the remaining children often had to find employment while being traumatized by losing their sibling, or had to drop out of school due to being discouraged about their uncertain futures. As a result, the parents were concerned about the other siblings, as to whether they would adjust in their social lives or get hurt from a privacy breach. They had become concerned with their children’s struggles with adjustment in school. Consequently, they expected intervening efforts for siblings of the student victims, for example support groups or a government-led rehabilitation plan for siblings of victims.

#### Relationships with co-workers, friends, and relatives

According to the study, since the Sewol ferry disaster, the victims’ families have experienced feelings of isolation and differences in their relationships with coworkers, friends, and relatives. Most struggled with feelings of discomfort and frustration when communicating with people they had known before the incident. They stated that it was difficult for them to express their feelings freely in front of people other than fellow victims’ families and that they avoid people who knew their circumstances:

More than anything else, we found it’s difficult to talk to other people. Actually, I could have tried harder, but just found myself avoiding it. You know, it’s even hard to talk to my family, then how is it not difficult talking with them? Together? (A 50-year-old father who lost a daughter)

They tried to tune into others around them in trying to get along. Yet the victims’ families reported feeling distant and that their acquaintances often approached them in nongenuine ways or showed mere sympathy. They often felt supported when the people around them demanded a thorough investigation of the Sewol incident, but felt discouraged when they were advised to go back to their homes and workplaces and stop their protests. Table 1 shows the changes in social relationships for the student victims’ families.

**Table 1:** Social relationship changes in student victims’ families after the Sewol ferry disaster.

Categories	Subcategories	Concepts
Relationships in proximal environment	Relationships with their immediate family	Changes in communication
		Increased sensitivity
		Changes in their families’ roles and structure
		Parents’ perceptions of other siblings’ states
		Parents’ concerns about other siblings
		Parents’ expectation about interventions for siblings
	Relationships with co-workers, friends, and relatives	Being different
		Being isolated
		Being rejected
		Discomfort
		Increased care for others
		Being supported
	Relationships with people related to the school	Conflict
		Concern for surviving students
Relationships in distal environment	Relationships with the community	Bonding
		Wariness
		Discomfort
	Relationship with citizens	Being supported
		Efforts to seek support
		Conflict and uneasiness
	Relationships with victims’ families committee	A sense of community
		Solidarity
		Within-group conflict
	Relationships with government and society	Fear
		Shame
		Distrust in the government and people in general
		Concerns about dysfunctional government
		Increased social concerns for people in pain

#### Relationships with the people related to the school

The victims’ families stated that they experienced much conflicts with people associated with the school. They had arguments over maintenance of classrooms, and also felt disheartened by surviving students and their parents who declined to participate in the victims’ families committee. They became reluctant to communicate with surviving students because their contact might pressure them:

We had another trauma coming. It’s because of the classroom. I moved to this town, decades ago, in 1994 right after I got married. So I know them really well. These people… they had a meeting once. I got there. Then, those people I’ve known for ten years, they shut me out and kept me from even entering the room. It’s shocking to me, honestly….(A 52-year-old mother who lost a son).

Although the families of student victims were struggling with the people associated with the school, they were also concerned about the well-being of the surviving students.

#### Relationships with the community

The victims’ families often felt bonded with their neighbors, but felt wary of them at the same time. They experienced a sense of being different and discomfort in their relationships with neighbors, so they often ended up mostly avoiding them:

If someone I know is coming this way, I lower my head and take a detour. It’s because I don’t want to see them. It was much worse at that time, than now. Shampoos, soaps, and something like that, they all were donated, and those things were very useful. It’s still the same. Yet, now I need some clothes or something, I just make an order on TV home shopping and have them delivered (a 47-year-old mother who lost a daughter).

#### Relationship with citizens

While the victims’ families reported that they felt supported by the public and also sought such support, they noted experiencing conflict and discomfort. They reported that civic groups, volunteers from the island Jindo, and oriental medical doctors had supported them enormously:

(The solidarity 416) I saw them on Facebook and got a chance to get there, Gumi last Saturday. It was so helpful just looking at how they worked (A 46-year-old mother who lost a son).

They sought support from the public and also tried to maintain a sense of solidarity with the public by holding meetings for public discussion and moderating their demands, taking into account public opinion. Despite their efforts, the families stated that they have struggled tremendously. For example, they reported incidents where they were slapped on the cheek and felt hurt by undervaluing statements about the Sewol ferry disaster. They reported uneasiness in that people seemed to misunderstand their compensation requests. They also felt sorry and worried about whether they were burdening other people or making them uncomfortable in general:

It’s not about monetary compensation. But, even yesterday, someone from the parents’ association talked to me. An old woman asked me, why we keep this tent and not stop protesting now that we got so much monetary compensation. She said we got that much money and should move on. Those kinds of words hurt us most, like cutting our heart out. We don’t accept the compensation money. We told them this so many times, but they don’t believe us. They said, “well, you got that much money; then why don’t you move on?” If you lost a child, can you forget and move on? It’s not them who lost a child. And they say things so easy (A 52-year-old mother who lost a son).

#### Relationships with the Sewol victims’ families committee

Many victims’ families reported that they struggled in daily life and had difficulty recovering their previous social relationships. Instead, they relied on a new community, the victims’ families committee. It was reported that among the student victims’ families, a strong bond and solidarity was formed so that they were able to share each other’s’ pain by supporting and caring for each other. They reported that, while working for the committee, they had shared personal tips for psychological coping and worked through their own suicidal urges and attempts:

Right! It helps each other. When we see other families, they’re the one who went through the same pain as ours. They are holing up too. I still think about killing myself. It’s still same that I got to follow (the child). But now, I have another strong feeling that I shouldn’t disappear like that (A45-year-old mother who lost a son).

The families had built up positive experiences by working for the families committee, but were also faced with conflicts regarding reparation and compensation for the incident. For instance, they often were discontented with and unconvinced about the work done by the committee, which they thought had made little progress in representing their plights and requests.

#### Relationships with government and society

The grieving families stated that they have struggled with feelings of anxiety about people and the world, and of bitterness about human nature and the unjust world, as a consequence of their changing social experiences after the Sewol ferry disaster. Some families were always anxious about the safety of their remaining children and nervous about seeing other people. Furthermore, the families felt shame about their dysfunctional government:

It’s even frightening to see other people, honestly. So, I don’t see anyone, though I have known them for decades. My brothers and sisters don’t come. Well, even though they come to see me, I try not see them. Because I can’t say nicely like before. Now I can’t be near anywhere people I know are around (A 48-year-old mother who lost a son).

Because of their changing social experiences after the incident, the families have experienced distrust in the government or people and growing worries about dysfunctional government. Their distrust had often developed due to suspicions about the official activities of the Korean national intelligence service or the Ministry of Oceans and Fisheries. For example, some had become paranoid that the government might track their locations or tap their cell phones. As one parent stated:

Really, even one little thing (doesn’t make sense). If the ferry didn’t leave port in such heavy fog, nothing would happen. Why they let it leave port, why let only the Sewol ferry leave? Why can’t the government investigate that? We’re really disappointed and frustrated. If the accident didn’t happen, we could just pull the ship up and find out why. If the government investigates how the ferry left port, everything will be clear. You know, honestly, I’m so frustrated about the government of South Korea, that can’t investigate anything. The way the government responded makes us more upset and angrier than the accident itself. Even if I went there [to Cheongwadae, an official residence of the South Korean president], would I shoot someone or do something bad? What would I do? Why don’t they just let us vent, instead of making us worse? They kept us from going forward in everywhere (A 46-year-old mother who lost a daughter).

And as another said:

The government doesn’t do anything to find out how it happened. In fact, it’s not that they can’t. It’s more like they don’t do it on purpose. Rather, the government, along with Cheongwadae and all institutions, is obstructing, covering up, and manipulating the truth. It’s really… (A 51-year-old father who lost a son).

Although they felt fear, shame, and distrust in the government and society, they have reported their increased care about people in pain since the Sewol ferry incident:

Now that I’m hurt, I could see people who were hurt. I could see the comfort women [the girls and women who were forced into sex slavery in the Japanese military during World War II. They were abducted from their home or lured by a promise of a job] in the Japanese military… Though it’s their pain, I could see their hurting now that I’m hurt. I would like to join them, and if there’s someone who’s hurt, I like to band together with them…. like I have been received. I don’t know what kind of job I will have, but I would like to help them by doing my job (A 22-year-old brother who lost a younger brother).

## Discussion

The analysis of the changes in social relationships for the Sewol victims’ families showed that they have experienced a variety of changes in their social relatedness. Changes in their social relationships were largely divided into relationships in the immediate/proximal environment and relationships in distal environments. The former included subcategories such as immediate family, coworkers, friends, relatives, surviving students, and the victims’ parents as well as concepts corresponding to each subcategory. The latter involved subcategories such as neighbors, other citizens, the victims’ family committee, government, and society as well as concepts corresponding to each subcategory.

First, the roles and characteristics of the immediate families have undergone considerable change since the Sewol ferry disaster. Whereas communication among family members had decreased in general, for some families, communication had increased. They stated that they have become more sensitive toward their own families. Parents expressed concerns about their remaining children and about how those children had changed. The grieving families struggled with the task of social adjustment due to recent significant changes in their surroundings, and with coping with multiple losses simultaneously. According to Dyregrov [20], when an individual loses a family member, he or she tends to struggle in daily life while being overwhelmed by long-lasting, strong feelings toward the lost one. Therefore, the remaining family members are likely to face several changes in their practical roles and the relationships associated with their roles and life structures. That is, the remaining family should search for their own ways of adjusting, while admitting that their loved one is gone. Their life goes through a long process in which they struggle to adjust to the new environment without the deceased.

While interacting with their close coworkers, friends, and relatives and also with the neighbors or general citizens, the victims’ families experienced feelings of being different, isolated, uneasy, and disappointed, although at times they felt supported by those same people. Moreover, it was found that they experienced various conflicts including arguments with surviving students and their parents over maintenance of the classrooms. According to Picou et al. [11], conflicts and competition tend to occur among victims, who are then likely to be exposed to rumors and to suffer from severe psychological anxiety and fear. In this study, it was reported that the victims’ families realized they were living in ‘a different world’ in their proximal and distal relationships and that such feelings had led them to avoid any acquaintance if possible. Normally, when an individual lacks interaction with his or her family, neighbors, friends, or people in a small network, a sense of isolation can occur [21]. Moreover, a parent who has lost a significant object of attachment, for example, a child, can suffer from difficulty relating with others [22], which in turn aggravates a sense of isolation. The grieving families felt frustrated with the surviving students’ parents whom they had once believed to be the most understanding. The conflicts with those parents again led the victims’ family to feeling isolated, and having the vicious cycle of avoidance and the following sense of isolation continue.

It was reported the victims’ families experienced fear, shame, and distrust in the government and society, and were also concerned the government was not working. As Erickson [23] indicated, if the party responsible for the disaster excuses themselves from culpability or evades their own accountability, victims and grieving families are likely to suffer from frustration and distrust of society and the world, which in turn can lead them to feel shame and humiliation. And their surrounding communities can be shattered into pieces beyond repair. Their painful experiences have led them to feel close to people who were hurt like they were. Such an increase in social consciousness for other marginalized groups serves as an example of how a sense of solidarity is constructed among the victims of social disasters during the course of mistreatment by the government.

Also, it was found that the grieving families found their greatest support from relationships with their fellow victims’ families who had also lost a child in the Sewol ferry disaster. The victims’ families had organized the families committee and built a strong bond, caring for and supporting each other. Such support shared by the families seemed to have helped them maintain mental health. Although at times they had conflicts with other families regarding coming up with a collective demand for compensation, they still were the most powerful resources for each other. It is known that social support can extensively moderate the impact of trauma, serving as a vital, protective factor for posttraumatic stress disorder [24–25]. Particularly in a collectivistic society, where social relationships and appraisals are highly appreciated, supportive interpersonal experiences can soothe the impact of a social trauma [26]. Similarly, the Sewol victims’ families depended on other victims’ families’ as offering the most reliable support each other.

The implication of this study’s findings can be summarized as follows. First, we examined the daily interpersonal experiences of the Sewol victims’ families, exploring how the disaster had changed their social relatedness. The results show that in the relationship with immediate family, parents were trying hard to be the parents of the lost child and of the remaining ones despite a significant change in their family morale and life styles. By contrast, it was found that in their general social interactions, except for those with the families committee, they experienced a sense of ' uneasiness' and 'conflict.' According to Doka [27], the mourning process would be stuck until the survivors could recognize the reality of or make sense of the loss. When parents who lost a child are unable to understand the reason and cause of the death, in their mourning they can suffer from great difficulties like severe adjustment problems [28]. It is likely that when the parents cannot make sense of the child's death, the child's psychological presence vividly goes on with the parents, while his or her physical presence disappears [29]. Although more than two years have passed since the disaster, the cause of and the incompetent rescue and search operation of the incident has not been fully investigated. Considering this predicament, the grieving family may have had difficulties accepting their loss and had developed a strong sense of distrust in society itself, which can bring about conflicts with various social relationships within the community.

Second, we focused on the social experiences of the parents of the Sewol student victims. Grieving the loss of a child entails intense emotional, behavioral, cognitive, and sociopsychological reactions [30]. Deeper exploration into such reactions is indispensable as effective rehabilitation from a manmade disaster needs to tap into subjective and individual feelings and perceptions of the disaster and of the reparation process, as well as the disaster’s explicit consequences.

Third, this study is intended to shed light on how to implement supportive social environments and aid for victims and their families suffering from a manmade disaster. According to the findings, the relationships with 'the families committee' made the victims' families feel supported and positive, through their ability to share their painful experiences and feelings during the incident with the fellow families [31], offering mutual support to each other. LaCapra [32], who studied the shooting at a Norwegian youth camp on the island of Utøya explained that the victims and their families were able to work through the pain of grief because the community as a whole shared their grief and painful memories while reflecting on the disaster altogether. Facilitating a family committee or small support groups can effectively promote experiences of mutual support and aid among victims and their families in that they can share common feelings and experiences about their loss and grief, with less concern about not being understood.

## Limitations

It should be pointed out that this study was conducted two years after the Sewol ferry disaster and that the families’ reported experiences may have changed over time. Therefore, a systematic assessment based on more detailed timelines could contribute to the establishment of long-term rehabilitation plans for the victims’ families. Also, there were some parents of the school student victims who did not participate in this study. It is likely that they might be more traumatized than other families. Those missing families are a clear limitation of this study. Also, the Sewol ferry victims' families are classified to two groups, the families awaiting recovery of missing bodies and the families who found the dead bodies. Although the research team gathered interview data from the families with missing children, the difficulties with those families were determined to be a significant subject requiring a whole different approach; therefore, the data was not used in this study. A future study can focus on the families of children of missing bodies, particularly on their coping with social conflicts, or explore further differences in changes in social relationships between the family who found the bodies and those awaiting recovery of bodies.

## Supporting information

S1 Appendix Semi-structured interview guide. (PDF)
