# Supplementary material for: Social relationship changes in victim families due to a social disaster: Experiences of student victims’ families in the South Korean Sewol ferry disaster
Source: PLoS One. 2017 Dec 7;12(12):e0188699. doi: 10.1371/journal.pone.0188699 (PMC5720753; doi:10.1371/journal.pone.0188699)
Supplement: S1 Appendix — (PDF) [file pone.0188699.s001.pdf]

Semi-Structured Interview Guide

The purpose of this study is to examine how social relationships of victims' families have changed since the Sewol ferry disaster. I am going to ask questions about your relationships with people and changes, if any, in these relationships during and after the catastrophe.

1. Describe your overall experience during and after the disaster.
    a. What was your initial reaction to hearing about the accident?
    b. What experiences have you had as you went through the grief process?
    c. How would you describe your interactions with people around you since the accident?

2. Describe your experiences with your immediate family members during and after the disaster?
    a. Please tell me about changes, if any, in your relationships with family members since the accident.
    b. What did you think or how did you feel about these changes?

3. Describe your experiences with relatives during and after the incident.
    a. Please tell me about changes, if any, in your relationships with relatives since the accident.
    b. What did you think or how did you feel about these changes?

4. Describe your experiences with your friends and acquaintances during and after the incident.
    a. Please tell me about changes, if any, in your relationships with people around you (e.g., friends, neighbors, co-workers, community members) since the accident.
    b. What did you think or how did you feel about these changes?

5. Describe your experiences with people who were involved in but you had not known well before the incident (e.g., survivors and their families, the committee of victims' families, civic groups).

6. Describe your experience in regard to aid and support from government and related

authorities?

7. What specific relationship(s) did you feel most helpful as you worked through the pain of grief? Please tell me how it has been helpful.

8. Any other thoughts or feelings that you would like to share?
